# Prevalence of mental disorders and torture among Tibetan refugees: A systematic review

**DOI:** 10.1186/1472-698X-5-7

**Published:** 2005-11-09

**Authors:** Edward J Mills, Sonal Singh, Timothy H Holtz, Robert M Chase, Sonam Dolma, Joanna Santa-Barbara, James J Orbinski

**Affiliations:** 1 Dept. of Clinical Epidemiology & Biostatistics, McMaster University, Hamilton, Canada; 2 Department of Medicine, Wake Forest University, Winston-Salem, USA; 3 Rollins School of Public Health, Emory University, Atlanta, USA; 4 Department of Community Health Sciences, University of Manitoba, Winnipeg, Canada; 5 Department of Political Science, York University, Toronto, Canada; 6 Department of Psychiatry and Peace Studies, McMaster University, Hamilton, Canada; 7 St. Michael's Hospital, University of Toronto, Toronto, Canada

## Abstract

**Background:**

Many Tibetan refugees flee Tibet in order to escape physical and mental hardships, and to access the freedoms to practice their culture and religion. We aimed to determine the prevalence of mental illnesses within the refugee population and determine the prevalence of previous torture reported within this population.

**Methods:**

We performed a systematic literature search of 10 electronic databases from inception to May 2005. In addition, we searched the internet, contacted all authors of located studies, and contacted the Tibetan Government-in-exile, to locate unpublished studies. We included any study reporting on prevalence of mental illness within the Tibetan refugee populations. We determined study quality according to validation, translation, and interview administration. We calculated proportions with exact confidence intervals.

**Results:**

Five studies that met our inclusion criteria (total *n *= 410). All studies were conducted in North India and 4 were specifically in adult populations. Four studies provided details on the prevalence of torture and previous imprisonment within the populations. The prevalence of post-traumatic stress disorder ranged from 11–23%, anxiety ranged from 25–77%, and major depression ranged from 11.5–57%.

**Conclusion:**

Our review indicates that the prevalence of serious mental health disorders within this population is elevated. The reported incidence of torture and imprisonment is a possible contributor to the illnesses. Non-government organizations and international communities should be aware of the human rights abuses being levied upon this vulnerable population and the mental health outcomes that may be associated with it.

## Background

In 1950, China began its occupation of Tibet. Since then, many Tibetans have fled to Nepal and India. More than 50 years of occupation have been accompanied by forced population displacements, widespread hunger, restrictions on cultural and religious freedoms, well-documented political violence against specific cultural groups, mass arrests, imprisonment of political prisoners and execution. Human rights groups have documented at least 60 deaths of peaceful demonstrators since 1987. [1] The current Tibetan population of Tibet is estimated at 6 million, with an undetermined number of Chinese occupants. This repression against Tibetans has resulted in large refugee populations in neighboring countries. It is estimated that more than 150,000 Tibetan refugees reside in the neighboring countries of Bhutan, Nepal, and India;[1] a generous token from such poor countries.

Due to political oppression, cultural oppression, and a desire to practice their religion, a growing number of Tibetans seek to escape to Tibetan settlements in India, the seat of the Tibetan Government-in-exile. The most common route is through Nepal. Depending on the point of departure and the type of transport used, the journey from Tibet to Nepal can take several days to months^1^. Most refugees cross the high mountains along commonly used escape routes to access Nepal. The Tibetan Refugee Transit Centre (TRTC), established by the United Nations High Commissioner for Refugees (UNHCR) in Katmandu, Nepal estimate that an average of 2,500 Tibetan refugees arrive into Nepal every year, with an equal number unsuccessful in their journey due to death or captivity. The TRTC assists refugees with their onward journey to India. The social background of those Tibetans who manage to flee has remained fairly constant over the past few decades, with a majority proportion of new arrivals being minors, monks and nuns. Nomads, farmers, and unemployed persons make up the remaining proportion. The number of attempted Tibetan refugee flights increases or decreases depending on the vigilance of Chinese border patrols.

Globally, at the start of 2004, there are an estimated 9.7 million refugees, included in a total of just over 17 million listed as 'persons of concern' for the UNHCR (including asylum seekers, internally displaced persons, returned refugees still being monitored, stateless persons, and refugees)[2]. Mental disorders are often overlooked in refugee populations. A recent analysis of mental illnesses within refugees residing in developed countries found that the prevalence of post-traumatic stress disorder (PTSD) was roughly 1 in 10, and major depressive disorder was roughly 1 in 20[3]. We previously examined the experiences of recent refugees in the flight from Tibet to Nepal and discovered that those interviewed were at a greater susceptibility for mental health issues than expected, due to traumatic events, torture, and unfamiliarity with their new surroundings[4]. The goal of this analysis was to review the prevalence of mental illnesses reported amongst the Tibetan refugee populations, and secondarily examine the reported incidences of torture by the Tibetan refugees. To determine this, we conducted a systematic review of the available literature.

## Methods

### Inclusion/exclusion criteria

Eligible studies assessed the prevalence of mental health disorders among Tibetan refugees in Nepal and India, and repatriated refugees in Tibet. Studies had to report original communications on the assessment of mental health outcomes using a measurement tool. We excluded qualitative studies, case reports and studies addressing the political experiences of participants. We additionally excluded studies assessing the mental health of Chinese nationals residing in Tibet.

With the aid of an information specialist, we searched the following 10 data bases (from inception to May 2005): AMED, CINAHL, Cochrane CENTRAL and the Cochrane library, EMBASE, ERIC, MedLine via PubMed, HEALTHSTAR, Psych-info, Sociological abstracts, and Web of Science. We additionally searched the internet using Google as well as bibliographies of relevant papers. Where appropriate we searched using the single term Tibet* as the yield was manageable. We contacted the authors of all studies for clarification of study outcomes, garnering a response rate of 100%. The Tibetan Government in Exile was contacted to determine if they were aware of unpublished research.

We used the definition of torture as stated in the United Nations Convention Against Torture and Other Cruel, Inhuman or Degrading Treatment and Punishment (UN Torture Convention)[5]. Torture, according to this convention, is defined as any act by which severe pain or suffering, whether physical or mental, is intentionally inflicted on a person for such purposes as obtaining from him or a third person information or a confession, punishing him for an act he or a third person has committed or is suspected of having committed, or intimidating or coercing him or a third person, or for any reason based on discrimination of any kind, when such pain or suffering is inflicted by or at the instigation of or with the consent or acquiescence of a public official or other person acting in an official capacity[5].

Two reviewers worked independently and in duplicate, to review the abstracts and full text versions of identified reports and to adjudicate their inclusion.

### Data abstraction

Two reviewers, working independently extracted data from the included studies using a standardized form. We specifically abstracted information on the following: population; duration of time outside of Tibet; number of children, clergy, elderly, and women; access to mental health services; and mental health outcomes (PTSD, anxiety and depression) identified using standardized measurement tools. We additionally extracted information on the number reporting imprisonment and torture.

### Methodological quality

In order to determine the validity of the interventions used to assess mental health status, we examined if these measurement tools had been translated and validated in the Tibetan language. We additionally assessed whether the tool was provided by interviewer administration or self-administered. We assessed whether reliability had been assessed and whether authors evaluated construct validity.

We determined the diagnostic criteria of the screening tools, where available, as follows: Harvard Trauma Questionnaire, scores above 2.5 are considered strongly suspicious of PTSD;[6] Hopkins Symptom Checklist-25, scores above 1.75 on the individual (anxiety and depression) of the HSCL-25 is consistent with significant emotional distress and correlates with the presence of diagnosable psychiatric morbidity. [7]

### Statistical analysis

Where proportions of populations were provided, we calculated the exact confidence intervals around the proportions. We did not pool results due to the heterogeneity of populations and methodologies employed. We tested interrater reliability using the K statistic. All statistics were performed using StatsDirect (Manchester, 2003).

## Results

Our systematic searches yielded 21 relevant abstracts. Thirteen were excluded as review articles. Of the remainder, 8 were selected for further examination and 4 were excluded as they were either qualitative or examined physical health outcomes. Four studies were included from academic publications[1,8-10]. One additional study was included from the non-government Organization (NGO) Physicians for Human Rights[11]. K for inclusion was ≥ 1, indicating perfect agreement. Table 1 describes the study populations. All studies were conducted in North India and all studies met the requirements of reporting specific methodological issues.

**Table 1:** 

Name, year	Setting; Country	Population	Design	N	Duration outside Tibet	Torture survivors (%)	Detained (n)	Measurement tools	Outcomes
PHR, 1997	Dharamsala Refugee Reception Center, Transit School for Young Adults, and a Buddhist monastery; India	Mixed population including clergy	Cross-sectional	258 (191 men and 67 women)	Median 6 months	21%	20%	10-item validated questionnaire to assess torture (UN Torture Convention)	15% (10–19%)
		Torture survivors		53		21%		HSCL-25-anxiety-depression	53% (40–66%) 40% (28–53%)
								DSM-IV criteria for PTSD	23% (13–39%)

Holtz, 1998		35 tortured Tibetan nuns and students with a cohort of 35 closely matched subjects.	Retrospective cohort	70	Mean of 30.9 months (SD = 19.2)	50%		For torture survivors HSCL-25 - anxiety - depression	54% (44–63%) 14% (8–22%)

Servan-Schreiber, 1998	Tibetan Childrens Village, Dharamsala, India	Children	Cross-sectional	61	Mean 13.3 months	NA	NA	DSM-IV PTSD DSM-IV Major depression	11.5 (3.5–19.5%) 11.5 (3.5–19.5%)
								DSM-IV Major depression	11.5 (3.5–19.5%)

Crescenzi, 2002	Confidential setting, Dharamsala, India	Purposeful adults -76 previously imprisoned 74 never imprisoned	Case-control	150	0–4 years	95%	50%	For total sample HSCL-25 - anxiety - depression HTQ assessment	63% (55–70%) 57% (49–65%) 20 (14–27%)

Terheggen, 2001	Refugee camp, North India	Random selection using random sequencing	Cross-sectional	76	1 month-2.5 years	12%	25%	PTI HSCL-25 - anxiety - depression	Median 3 (range 0–3) 25% (17–36%) 42% (32–53%)

HSCL, Hopkins Symptom Check List, PTI, Post Traumatic Inventory

Three studies reported on PTSD (total *n *= 264) [1,8,11]. The prevalence of post-traumatic stress disorder ranged from 11–23%. Four studies reported on prevalence of an anxiety disorder (total *n *= 349) and ranged from 25–77% [8-11]. Five studies reported on prevalence of major depressive disorder (total *n *= 357)[1,8-10] and ranged from 11.5–57%.

Below we report on the individual studies and include details on the study population; incidence of torture, and; prevalence of mental disorders, respectively.

### Physicians for Human Rights (PHR)

#### Study population

Physicians for Human Rights[11] (PHR) conducted a large convenience sample survey using a validated checklist to assess the prevalence of torture and imprisonment among newly arrived Tibetan refugees in Dharamsala, India. Fifty-five individuals reported being tortured (21%). Those who were reportedly tortured tended to be young at their first episode of torture (mean age 19.5, range 13–28). Fifty-eight percent (32/55) were less than 21 years old, and 15% (8/55) were 16 years old or younger at the time of their torture.

#### Incidence of torture

Sixty percent (33/55) of the torture victims in this study reported being subjected to three or more different forms of torture in addition to threats or verbal abuse. Torture by electric shock commonly employed the use of cattle prods, including applying the cattle prods to the genitals, mouth and eyes. One individual reported being immersed in a tub of water, before being forced to lay down on an electrified metal bed. Two forms of torture that PHR identified, which previously have been infrequently reported, included being forced to stare at the sun for prolonged periods of time, and having blood drawn against the individual's will.

#### Prevalence of mental disorders

PHR further examined mental health outcomes within the torture survivors subgroup by utilizing the Hopkins Symptom Checklist (HSCL-25) to assess emotional distress and the DSM-IV criteria to diagnose PTSD. Seventy-eight percent of the torture survivors to whom the Hopkins Symptoms Checklist-25 was administered were suffering from significant symptoms of anxiety and/or depression. Eighty-eight percent of the torture survivors surveyed concerning PTSD symptoms reported recurrent, intrusive memories, including flashbacks and nightmares of their abuse. Twenty-three percent of these individuals met DSM-IV criteria for PTSD.

### Holtz

#### Study population

A retrospective cohort study was conducted in Dharamsala India in 1995[9]. Thirty five refugee Tibetan nuns and lay students who were arrested and tortured in Tibet were matched with 35 Tibetan refugee controls not previously arrested or tortured. The groups were similar in most respects, with the exception that the tortured group had been more politically active than the controls, whilst living in Tibet. The mean age of the torture survivors was 25.8 years(SD = 2.3 years).

#### Incidence of torture

The torture events took place during a mean of 21 months of captivity. The mean length of abuse was 38 days. Fifty-seven percent (20) reported solitary confinement, with a mean confinement period of 5.4 weeks. Overall, the survivors reported exposure to a mean of 13.3 different forms (SD = 3.2) of torture, ranging from 7 to 21 types. Only 43% (15) of those tortured were ever charged with a crime, and fewer (7%) actually brought to a trial. One survivor remembered the presence of a doctor while being tortured. Forms of torture characteristic of Chinese prisons in Tibet include electrical shocks to the body (86%), forced standing (86%), exposure to bright sunshine (69%), and having blood extracted without consent (50%).

#### Prevalence of mental disorders

Participants were administered the HSCL-25, to evaluate anxiety symptoms, affective disturbances, somatic complaints, and social impairment. The prevalence of symptom scores in the clinical range for both cohorts was 41.4% for anxiety symptoms and 14.3% for depressive symptoms. The torture survivors had a statistically significant higher proportion of elevated anxiety scores than did the nontortured cohort(54.3% *vs. *28.6%, *p *=.01). Depressive scores were not different between groups. The results suggest that torture has long-term consequences on mental health over and above the effects of being uprooted, fleeing one's country, and living in exile.

### Servan-Schreiber

#### Study population

A cross-sectional study examining refugee children in Dharamsala India[1], was conducted to asses PTSD and depression within a Tibetan children's population. Sixty one randomly selected children from the Tibetan Children's Village School were administered a questionnaire by a psychiatrist assessing DSM-IV diagnosis for PTSD and major depression. The average children's age was 12.6 (SD 2.5) and was equally representative of males to females (30 vs. 31).

#### Prevalence of mental disorders

PTSD was diagnosed in 11.5% of the children (95% CI, 3.5–19.5%), with a further 18% with suspected PTSD (95% CI, 8.4–27.4%). There was a trend for more cases of full criteria PTSD amongst children who had recently arrived (<18 months) (25% vs 6.7%, chi-square, *P *= 0.06). Major depression was diagnosed in 11.5% of the population with 18.9% of all children over the age of 13 meeting the full criteria for major depression. The study did not report the proportions or quantifiable number of children who had experienced torture or imprisonment, but did include children who had been physically traumatized.

### Crescenzi et al

#### Study population

In a case-control study in Dharamsala, India[8], Crescenzi et al. compared mental health disorders among 76 previously imprisoned refugees to 74 never imprisoned refugees. Forty-five percent of the total sample had been clergy in Tibet. All participants were >18 years of age at the time of escape from Tibet. Utilizing a detailed translation of the HSCL-25 checklist, the authors assess anxiety and depression, as well as listed traumatic events.

#### Incidence of torture

Although the previously imprisoned group had a higher incidence of torture, most participants in both groups had experienced some degree of torture. The most common torture techniques across both groups were beatings (73%), electrical torture (43%), being forced to provide blood (19%), and being kept naked (25%). In addition to torture, both groups reported traumatic events which may affect mental health. These included sleep deprivation (36%), witnessing murder (37%), kidnapping of family and friends (37%), and disappearances of family and friends (13%).

#### Prevalence of mental disorders

The authors found that refugees who were previously imprisoned were more likely to experience mental health issues than those never imprisoned, although both groups had elevated mental health symptoms [HSCL-anxiety scores 22.5 (SD = 6.5) vs. 18.7 (SD = 6.8), P = 0.001] [HSCL-depressive scores 28.5 (SD 7.4) vs. 27.6 (SD 7.4), P=>0.05]. PTSD was diagnosed in 20% of the total sample and anxiety and depression were diagnosable amongst 63% and 57% of the total sample, respectively.

### Terheggen et al

#### Study population

In a cross-sectional study of refugee students residing at a North India refugee camp[10], Terheggen et al. interviewed 75 individuals about past traumatic events and assessed the correlation between traumatic events and severe mental health diagnosis. Participants were new refugees and ranged in age from 18–29 years. Sixteen percent made weekly visits to their physician.

#### Incidence of torture

The issues identified by the participants as most traumatic included: destruction of religious signs (43%), relative or friend tortured (28%), relative or friend disappeared (26%), relative or friend sent to prison (28%), being tortured oneself (12%), being sent to prison (25%), and fearing for one's own life (32%). In addition, important traumatic events were identified that were unexpected, such as: being forbidden to speak own language (3%), being forbidden to live according to one's own religion (15%), and being forbidden to live according to one's own culture (9%).

#### Prevalence of mental disorders

The study found a high prevalence of anxiety (25%) and depression (42%) among the population. Importantly, the study found a correlation between incidences of trauma and mental health diagnoses (0.67). This study is the only study which identified the trauma that the refugees perceived when witnessing the destruction of the religious artifacts.

## Conclusion

The findings of this systematic review should be of interest to NGOs, policy makers and politicians. We found that the prevalence of mental illnesses within the Tibetan refugee population is remarkably high, with increased incidences in the many refugees reporting severe torture and violations of human rights. Governments and NGOs should be aware that human rights violations are impacting mental health outcomes in this vulnerable population.

There are certain limitations to interpreting our study. We performed a systematic review using standard searching techniques. It is possible however, that NGO reports were not published and as such, were excluded without our knowledge. However, we did consult the Tibetan Government in Exile for their knowledge regarding additional studies and none were located. A further limitation is that not all studies examined the impact of torture on mental health illnesses, and it is possible, and likely, that torture victims have increased susceptibility to mental illness, such as PTSD. Studies used diverse instruments with which to determine the prevalence of mental health illnesses. In the studies we included, 4 used screening tools to evaluate anxiety and depressive disorders [8-11] Only 1 consistently used diagnostic tools.[1] The accurate assessment of psychiatric disorders is difficult to ensure in epidemiological studies, especially in non-Western populations, for whom the validity of measures developed in Western populations may be restricted[3,12-14]. In our review, most assessments of anxiety and depression were determined by the Hopkins Symptom Check List (HSCL), a tool with extensive use internationally[15]. The HSCL-25 is the most commonly used scale to assess anxiety and depressive symptoms amongst refugee populations. Translations of the HSCL-25 have been validated against clinically-assessed diagnoses of anxiety and major depression[7], for use in several south Asian refugee groups[7,16,17], but have not tested for construct validity in a Tibetan population. Recent translations of the HSCL-25 into a variety of languages have demonstrated good psychometric properties and have been widely used in adolescents and adults to assess psychiatric morbidity in traumatized populations and refugee groups worldwide[12,18-20] The test-retest reliability of this instrument has been found to be r = .90 for Southeast Asian refugees[21]. The diagnosis of PTSD was however, varied. Two studies used DSM-IV diagnostic case tools[1,11], while 1 used the Post-Traumatic Inventory (PTI)[10] and 1 used the Harvard Trauma Questionnaire[8], a well-validated questionnaire with good correlation with clinical diagnosis. The studies examined were all conducted in North India, after the refugees had arrived from Nepal. It may be that the incidence of mental health illnesses are higher during their stay in Nepal and decrease with time, as was observed in several included studies[1,8,9]. Further, the studies included in our review were generally limited in methodological quality. No study assessed test-retest reliability or construct validity. Only one study used a random-sampling technique[10] All studies were limited in their sample size and it is possible that this contributes to a sampling bias leading to a higher incidence.[22] A final limitation is that this study only examined incidents of torture reported by the refugees. Other Tibetans who are 'persons of concern' for the UNHCR are also likely to have experienced torture, and were not included for analysis in this review.

Our findings indicate that the prevalence of mental health illnesses within this population are higher than those reported in most refugee populations[3,22]. Of particular concern is the high prevalence of PTSD and depression among the children examined[1]. The Tibetan Government in Exile should be urged to encourage children to report on violations and provide specialized treatment programmes for children. These findings are consistent with the findings of a previous qualitative study indicating that children are often witnesses and victims of human rights violations and exploitation, including sexual exploitation[4].

Our findings demonstrate that torture is commonly reported amongst Tibetan refugees, and that those who have experienced torture often suffer significant psychological effects. The findings in this study have a number of important implications for the health of the Tibetan refugee community and the international community as well. The Tibetan Government in Exile provides a specialized torture treatment program only for political torture survivors [23]. Access to care for all survivors of torture, both political and non-political, needs to be provided, as well as specialized programs for children. In addition, this study illustrates the paucity of generalizable information about the mental health of this population in what appears to be a sustained assault on the health and dignity of the Tibetan culture and community. In our review, we found that severe torture, including electric cattle prods on genitals and oral cavities, as well as forced blood draws, was routinely reported. In addition, many refugees cited their mental health illnesses as stemming from witnessing their family and friends murdered.

Under the Criminal Law of the People's Republic of China, it is forbidden to extort a confession by torture[24]. Furthermore, China is a signatory to a number of international human rights conventions, legally binding under international law, including the Convention Against Torture and Other Cruel, Inhuman or Degrading Treatment or Punishment[25], and the Convention on the Rights of the Child[26]. Based upon our systematic review, wherein all systematic torture and detainment occurred in China, China is in clear violation of these conventions. [11]

All governments and other economic partners to China should be aware that human rights violations are unacceptable, and should pressure the Chinese government for adherence to international human rights and humanitarian standards, including prohibitions against the use of torture. In order to prevent further illness and human rights violations, international NGOs should bring pressure to bear on the Chinese government to ensure independent and impartial access to political and cultural prisoners. NGOs working in Nepal and North India should be aware that mental health illnesses are prevalent within this community and make efforts to deliver care and ensure appropriate political representation for the displaced individuals. Governments should be aware that human rights violations are unacceptable behavior from an economic partner, and pressure the Chinese government for change. Finally, the Tibetan Government in exile must recognize that mental health illnesses are widespread within the refugee populations and reach out to sympathetic nations for appropriate assistance.

## Competing interests

The author(s) declare that they have no competing interest.

## Authors' contributions

Concept: EM, SS, TH, SD, JSB, JO

Data searching: EM, SS, TH, SD, JSB

Analysis: EM, SS, TH, SD, JSB, JO

First draft: EM, SS, TH, SD, JSB, JO

Critical revisions: EM, SS, TH, SD, JSB, JO

## Pre-publication history

The pre-publication history for this paper can be accessed here:


